# Using Arts-Based Therapies to Improve Mental Health for Children and Young People With Physical Health Long-Term Conditions: A Systematic Review of Effectiveness

**DOI:** 10.3389/fpsyg.2020.01771

**Published:** 2020-09-25

**Authors:** Sarah Wigham, Patricia Watts, Ania Zubala, Sharmila Jandial, Jane Bourne, Simon Hackett

**Affiliations:** ^1^Faculty of Medical Sciences, Population Health Sciences Institute, Newcastle University, Newcastle upon Tyne, United Kingdom; ^2^Teapot Trust, Edinburgh, United Kingdom; ^3^Institute of Health Research and Innovation, University of the Highlands and Islands, Inverness, United Kingdom; ^4^Newcastle upon Tyne Hospital NHS Foundation Trust, Newcastle upon Tyne, United Kingdom; ^5^Cumbria, Northumberland Tyne and Wear NHS Foundation Trust, Newcastle upon Tyne, United Kingdom

**Keywords:** pediatric, arts-based therapies, physical health long-term conditions, mental health, children

## Abstract

**Background:** Children with physical health long-term conditions (LTCs) have increased risk of mental health difficulties relative to healthy peers. However, availability of psychological support integrated into pediatric physical health settings is limited, and there are long waiting times for access to child mental health services. Arts-based therapies involve using creative media to develop a therapeutic relationship, and offer a potential alternative to talking-based therapies. The aim of this systematic review is to establish the effectiveness of arts-based therapies for improving the mental health of children with physical health LTCs.

**Methods:** The review protocol was published on PROSPERO. Four electronic databases were searched (Medline, Embase, Cinahl, and PsycINFO), plus hand searches of two key journals and relevant reviews, and forward/back citations searches of selected articles were conducted. The Effective Public Health Practice Project (EPHPP) Quality Assessment Tool was used to assess bias in selected articles. Second reviewers completed 10% of article screening and 20% of bias assessments. The findings were synthesized narratively.

**Results:** Sixteen studies met inclusion criteria and demonstrated some improvements on indicators of mental health and well-being including quality of life, coping behaviors, anxiety, self-concept, and mood. However, replication across interventions and outcomes was absent. Overall, the quality of evidence of effectiveness in the studies reviewed was moderate/weak. This was due to bias in study design; other limitations included a lack of detail on intervention components, e.g., use of a manual, and single recruitment sites.

**Conclusions:** The heterogeneity of existing research evaluating arts-based therapies for children with physical health LTCs limits conclusions about effectiveness. Suggestions are made to inform the design of future research studies to help build a robust evidence base.

## Introduction

Pediatric physical health long-term conditions (LTCs) have a childhood onset, require ongoing management over a long period of time, are often multi-system, and typically require complex treatments with both medications and non-pharmacological interventions and services (Mokkink et al., 2008; DoH, 2012). Pediatric physical health LTCs require significant psychological adjustments to symptoms, medical regimes, and pain and may detrimentally affect the well-being of children relative to healthy peers (Denny et al., 2014; Kazak et al., 2015). Children may be isolated from peers during hospital stays and miss participating in activities that contribute to quality of life, and research suggests that children with physical health LTCs are susceptible to poor mental health (Knight et al., 2015; Butler et al., 2018). The prevalence of anxiety and depression is high, and mental health and well-being difficulties are up to four times higher in children with physical health LTCs than in healthy peers (Hysing et al., 2007; Pinquart and Shen, 2011a,b; Ferro, 2016; Brady et al., 2017). Mental health is a key predictor of a successful clinical course; for example, baseline depression in children with rheumatic diseases was associated with higher levels of pain and disability 4 years later (Colver et al., 2018; Gray et al., 2018; Hanns et al., 2018). Good childhood mental health optimizes engagement in daily activities for children and families; facilitates psychosocial development, educational attainment, and increased life-course opportunity; and protects against adult mental health problems (DoH, 2011; Colver et al., 2018; Tollisen et al., 2018). Additionally, parents of children with physical health LTCs are prone to high levels of stress/distress, and poor mental health of their child may be an additional burden on them and on child mental health services, which are under-resourced (Crouch et al., 2019; Rosenberg et al., 2019).

Given the potential burden of poor mental health, UK statutory guidance identifies integration of psychological support into pediatric physical health settings as an indicator of service quality (DoH, 2011; Foster et al., 2017; Parsons et al., 2017; NHSE, 2018). However, evidence suggests that services for pediatric physical health LTCs are not routinely set up in this way, staff time and resources are limited, access to psychological support is patchy, and there are long wait lists for child and adolescent mental health services (Wiener et al., 2015; Cruikshank et al., 2016; Davis et al., 2017). Further, there are currently significant gaps in the evidence base for the best type of psychological support for children with physical health LTCs (Kazak et al., 2015; Knight et al., 2019).

A review evaluating psychological interventions for anxiety and depression in children with physical health LTCs found cognitive behavioral therapy (CBT) effective under certain circumstances—in the short-term for mild/moderate symptoms of depression (Thabrew et al., 2018). A recent evidence synthesis evaluated a range of mental health interventions for children with physical health LTCs, for example, parenting interventions, play therapy, relaxation, and emotional intelligence training (Moore et al., 2019). The review utilized meta-ethnography and identified benefits from the perspectives of patients, families, and practitioners experiencing the interventions (Moore et al., 2019; Shaw et al., 2019). The authors developed a conceptual model of constructs (e.g., empowerment) important for enabling benefit from mental health interventions for children with physical health LTCs (Moore et al., 2019; Shaw et al., 2019). Although some evidence of effectiveness was found for CBT, the review also highlighted an overall lack of quantitative evidence of the effectiveness of mental health interventions for children with physical health LTCs and the absence of trials conducted in the UK (Moore et al., 2019).

The aim of the current review is to inform preparation for a feasibility trial of arts-based therapies in the UK National Health Service (NHS). NICE defines arts-based therapies as psychotherapeutic techniques combined with creative activities to facilitate self-expression and recommends their provision in the management of children with psychosis (NICE, 2016). Arts-based therapies involve using creative media to develop a therapeutic relationship and can be an alternative to talking-therapies through facilitating the expression of inner states that are difficult to articulate verbally (American Art Therapy Association, 2018; Fancourt and Finn, 2019). Although the benefits of arts-based therapies in mental health settings suggest the potential for transferability to physical health services, arts-based therapies are not routinely available as a psychological support in UK NHS physical health settings.

Research has demonstrated some positive effects of arts-based therapies for children with physical health LTCs; however, the evidence base is small (Cohen-Yatziv and Regev, 2019). Previous reviews relevant to this clinical group include two reviews of visual art therapy—one for children with a range of physical health conditions (Clapp et al., 2018) and one focusing on children with cancer (Aguilar, 2017). A systematic review of music interventions for patients with cancer was not specific to children (also included adult samples) and included studies evaluating physical health outcomes (without measurement of mental health outcomes) and studies evaluating music as a distraction (Bradt et al., 2016). These reviews identified some small evidence of effectiveness and some bias in the design of previous studies, and our review builds on the findings of these reviews. Our specific focus is evaluating the potential of arts-based therapies as a psychological support for pediatric physical health settings and the identification of methodological characteristics important to consider in designing future robust research studies to contribute to building an evidence base (Cohen-Yatziv and Regev, 2019). In preparation for a feasibility trial of arts-based therapies in the UK NHS, we reviewed existing arts-based therapy studies in order to help design the trial.

### Aims of the Study

The aims of this systematic review are to (i) identify studies evaluating the effectiveness of arts-based therapies for improving the mental health of children with physical health LTCs and (ii) examine the quality of the identified studies using a formal assessment tool.

## Methods

A systematic review was reported in accordance with PRISMA guidelines (Preferred Reporting Items for Systematic Reviews and Meta-analyses) (Moher et al., 2009). The protocol was published on PROSPERO (CRD registration number: 42019134461).

### Inclusion Criteria

Inclusion criteria for the review and PICO components (population, intervention, comparator, and outcome) are shown in Table 1A. Inclusion criteria comprised the following: studies recruiting participants ≤18 years of age with pediatric physical health LTCs, defined as requiring ongoing management with medication and/or other treatment interventions over a long period of time (Mokkink et al., 2008; DoH, 2012); studies evaluating arts-based therapies, defined as systematic interventions implemented by a therapist with the aim of improving health through creative expression (for example, drawing, play, photography, movement, music) and a therapeutic relationship promoting communication, connection, and self-awareness (NICE, 2016; Hackett et al., 2017; American Art Therapy Association, 2018); additionally, only articles published after the year 2000 given rapid developments in medical settings and treatment interventions; and finally, those using a published outcome measure or structured assessment (e.g., time sampling) of mental health or well-being.

**Table 1A:** Inclusion criteria.

Inclusion criteria	Population: children (participant mean ≤18 years of age) with physical health long-term conditions (LTCs) Intervention: arts-based therapies Comparator: standard care, none, before/after Outcome: change in mental health symptoms or psychological well-being measured using either (i) an assessment tool (for which published information/psychometric properties is available) or (ii) a formalized assessment method, e.g., time sampling Study design: quantitative Articles published in English since 2000
Exclusion criteria	Babies; passive arts interventions (for distraction or improving skills); unpublished documents; case studies Studies reporting only on mobility, communication, or cognitive functioning; studies combining arts-based therapies with cognitive behavioral therapy

We conducted the searches in May 2020, and Table 1B shows the information sources. We searched four electronic databases, conducted hand searches of two key journals, searched reference lists of relevant reviews, and conducted forward/back citations searches of articles meeting inclusion criteria. The search strategy for Medline is shown in Table A1.

**Table 1B:** Information sources.

Scoping searches	Health and Social Care Information Center Health Management Information Center Cochrane Library [Cochrane Database of Systematic Reviews, Cochrane Central Register of Controlled Trials (CENTRAL), Cochrane Methodology Register, Health Technology Assessment Database]
Formal searches	Electronic databases searched: MEDLINE, EMBASE, PsycINFO, CINAHL
Other sources searched	Reference lists relevant systematic reviews; forward and back citation searches of included studies; Google search of search terms; hand searches in: *International Journal of Art Therapy; Arts and Health. An International Journal for Research, Policy and Practice*

### Study Selection

**Figure 1:**
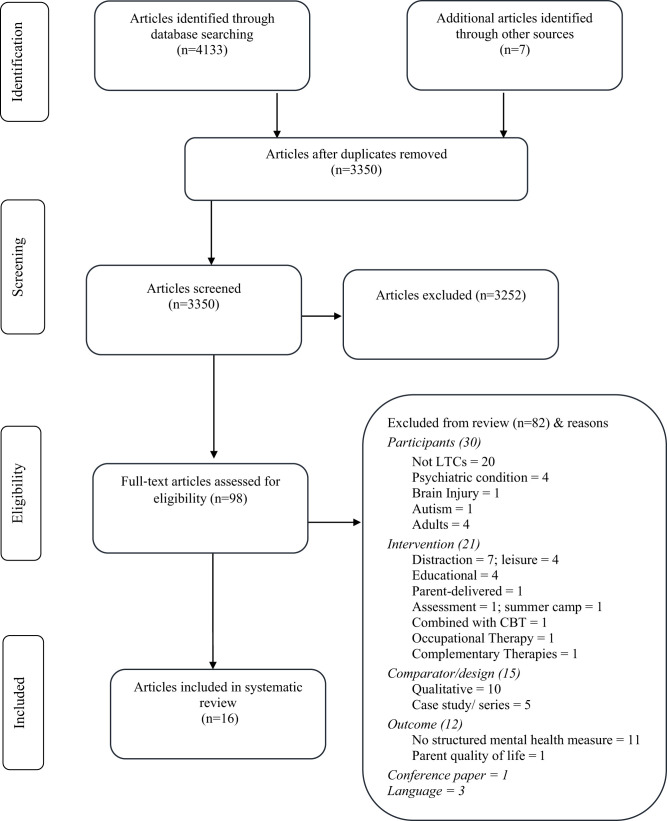
The PRISMA flowchart showing study selection process.

One researcher (SW) screened titles and abstracts of articles identified in the searches, with 10% independently screened by a second reviewer (AZ), and the level of agreement was 96%. Ambiguous articles were included. 98 articles were selected for full-text screening. SW carried out full-text screening, with 10% screened independently by a second reviewer (AZ). Agreement was 100%. Figure 1 shows the selection process to determine the eligibility of articles for inclusion in the review.

### Synthesis

Due to high heterogeneity in study design, quantitative synthesis of information was not possible, and we used a narrative synthesis of intervention effectiveness incorporating four elements—intervention conceptualization and theoretical model, tabulation of information, exploring patterns within and across studies, and assessment of methodological quality (Popay et al., 2006).

### Intervention Conceptualization and Theoretical Model

Various conceptualizations of arts-based therapies occur in the literature. In this review, we focus on arts-based therapies (visual, drama, music, and play) that include interaction, development of a therapeutic relationship, and facilitating communication and expression (NICE, 2016; Hackett et al., 2017; American Art Therapy Association, 2018). We excluded articles evaluating use of art media for distraction, (e.g., while having an injection), for leisure (e.g., listening to music), or for education. The purpose of the review was to inform the design of a randomized feasibility study of arts-based therapies in the UK NHS. We built a logic model (Figure 3) showing areas we felt may be important in a trial evaluating the effectiveness of arts-based therapies in pediatric physical health settings in order to guide information extraction and interpretation of the review findings, and we developed the model informed by the synthesis (Popay et al., 2006).

### Assessment of Methodological Quality

Studies were assessed for risk of bias by SW using the Effective Public Health Practice Project (EPHPP) Quality Assessment Tool (Thomas et al., 2004). JB and AZ independently rated 20%, and agreement was 80%.

## Results

### Key Characteristics of Studies

Three thousand three hundred and fifty articles were identified from the electronic database searches after removal of duplicates. After title/abstract screening, 98 articles were screened in full, and 16 articles were selected for inclusion in the review. Information was extracted and tabulated, with Tables 2, 3 showing study characteristics and Table 4 showing significant findings from the selected articles.

**Table 2:** Participant, study, and intervention characteristics.

**References (country)**	**N (F)**	**Age (years) mean (SD); range**	**Recruitment source**	**Health condition**	**Design *Control***	**FU (month)**	**Intervention media**	Attrition^*^
**Children with cancer: visual media**								
Khodabakhshi Koolaee et al. (2016) (Iran)	30 (16)	10.3 (NR); 8–12	Oncology unit	Leukemia	CCT *TAU*	1	Visual art	NR
Abdulah and Abdulla (2018) (Iraq)	60 (25)	9.5 (2); 7–13	Oncology unit	Heterogeneity of malignancies	RCT *TAU*	0	Visual art	1
**Children with cancer: music media**								
Robb (2000) (USA)	10 (NR)	NR; 4–11	Oncology isolation	Cancer	Within-subjectdesign	0	Music	NR
Barrera et al. (2002) (Canada)	65 (33)	7 (4.8); 0.5–17	Hematology oncology unit	Heterogeneity of malignancies	Cohort	0	Music	5
Colwell et al. (2005) (USA)	24 (9)	12 (NR); 7–18	Inpatient unit	>75% cancer	CCT *TAU (art)*	0	Music	0
Robb et al. (2008) (USA)	83 (NR)	(NR); 4–7	Inpatient oncology 6 sites	Cancer	RCT *music listening;* *audio-storybooks*	0	Music	NR
Giordano et al. (2020) (Italy)	48 (NR)	(NR); 2–13	Oncology and hematology ward	Cancer	CCT *TAU*	0	Music	NR
	19		Medical staff					
**Children with cancer: composite arts-based therapies (music, movement, and art) and virtual reality play therapy**								
Madden et al. (2010) (USA)	18 (4)	5.3 (NR); 2–13	Outpatient oncology; chemotherapy infusion	Cancer	RCT *Attention*	0	Music, movement and visual	2
	32 (14)	8.3 (NR); 3–21			Cohort	0		
			Service providers		Focus groups	12		
Li et al. (2011) (Hong Kong)	122 (57)	12 (2); 8–16	Pediatric oncology ward	Cancer	Quasi-experimental *TAU*	0	Virtual reality play	8
**Children with blood disorders and other health conditions: visual media**								
Beebe et al. (2010) (USA)	22 (NR)	NR; 7–14	Specialist school clinic	Asthma	CCT *Wait list*	6	Visual art	0
Stafstrom et al. (2012) (USA)	16 (10)	12.8 (2.9); 7–18	Pediatric neurology clinics	Epilepsy	Cohort	0	Visual art	NR
MacDonald et al. (2019) (Canada)	12 (10)	17.75 (NR); 15–21	Provincial diabetes program	Diabetes	Cohort	0	Visual art	5
**Children with blood disorders and other health conditions: music media and play therapy**								
Colwell et al. (2013) (USA)	32 (17)	11 (3); 6–17	Inpatient unit	Cancer, sickle-cell disease, accidents, infections	CCT (3 groups) Music listening and composition	0	Music	NR
Robb et al. (2014) (USA)	113 (47)	11–24	Multi-site (8 sites)	HSCT	RCT *Audio-books*	3	Music	NR
Tomaj et al. (2016) (Iran)	60 (30)	9.50 (1.74); 7–11	Thalassemia units 2 hospitals	ThalassemiaMajor	RCT *TAU*	1	Play	2
Uggla et al. (2018) (Sweden)	29 (15)	0–17 (6.6)	Hospital	HSCT	RCT *Wait list*	6	Music	9

*N, number of participants; F, female; CCT, controlled clinical trial; NR, not reported; RCT, randomized controlled trial; C, control; TAU, treatment as usual; FU, follow-up; HSCT, hematopoietic stem cell transplant*.

* *Intervention group*.

**Table 3:** Intervention characteristics.

**References**	**Number of sessions; intervention length**	**Individual (I) group (G)**	**QI**	**Intervention**
**Children with cancer: visual media**				
Khodabakhshi Koolaee et al. (2016)	2 per week (1 h); 11 sessions	NR	Y	Painting, collage, drawing
Abdulah and Abdulla (2018)	20 (2 h); 1 month	G	Fine artist	Drawing and craftwork. Encouragement of reflection and description of art work (cardboard, wood, watercolors, markers).
**Children with cancer: music media**				
Robb (2000)	1 h	I	Y	Control; reading; music; control (15 min each) Intervention schedule: 4–7 and 8–12 years versions
Barrera et al. (2002)	1–3 (15–45 min)	I and family	Y	School age: singing and song writing, improvisation and music listening Pre-school: animated play songs, rhymes, playing instruments Infants: play, songs, lullabies, rhymes, playing instruments. *Instruments: e.g., bells, drums, shakers, guitar, electronic harp/keyboard, songbooks, means of recording and playing music*
Colwell et al. (2005)	45 min	I	Y	Music computer program: CD composition and creation
Robb et al. (2008)	20 min	I and parent	Y	Active music engagement (AME) (music, acoustic guitar, hand instruments, illustrated songbooks, puppets, toys): playing instruments action/illustrated songs. Intervention guide used.
Giordano et al. (2020)	15–20 min 1–6 sessions	I and parent	Y	Individually tailored interactive relational approach with active and receptive techniques, use of musical instruments, improvisation, singing, song writing, creation of/listening to music with the therapist
**Children with cancer: composite arts-based therapies (music, movement, and art) and virtual reality play therapy**				
Madden et al. (2010)	Randomized group: weekly (1 h ×6) 2 sessions in each CAT modality	I	Y	CAT (movement, music, art) replicated developmental expression from movement, sound, graphics (for each patient in same order)
	Cohort: 1 h session	G		
Li et al. (2011)	30 min × 5 days a week	G	Research nurse	Virtual reality game
**Children with blood disorders and other conditions: visual media**				
Beebe et al. (2010)	7 × 1 h sessions; 7 weeks	G	Y	Discussion, art-making, sharing feelings. Intervention schedule provided.
Stafstrom et al. (2012)	4 × 1.5 h sessions; 1 month	G	Y	Drawing, painting collage and digital
MacDonald et al. (2019)	12 × 90 min; weekly	I and closed G	N	Relaxation, activities to develop self-awareness, trust, respect Drawing, painting, collage, paper sculpture, clay, fabric, found objects Theoretical model: existential, person-centered, and cognitive behavioral Intervention schedule provided
**Children with blood disorders and other conditions: music media and play therapy**				
Colwell et al. (2013)	1 × 45 min	I	Y	Orff-based approach: active music making, rhythmic book reading (*Hooray for You! A Celebration of You-ness*) talking about self and goals
				
Robb et al. (2014)	6 sessions (2 per week)	G	Y	Therapeutic music video (TMV)
Tomaj et al. (2016)	8 × 45–60 min sessions over 1 month	G	Researcher	Playdough, clay, mud, storytelling, and coloring
Uggla et al. (2018)	45 min × twice a week for 4–6 weeks	I	Y	Singing, music playing/listening. Parents could participate.

*NR, none reported; CAT, creative art therapy; N, no; Y, yes; QI, qualified interventionist*.

**Table 4:** Outcome measures and results.

**References**	**Outcome measures**	**Results**
**Children with cancer: visual media**		
Khodabakhshi Koolaee et al. (2016)	Children's Inventory of Anger (Nelson and Finch, 2000) Spence Children's Anxiety Scale (Spence et al., 2003)	Significant pre/post-intervention reductions in anger (*p* = 0.001), anxiety (*p* = 0.001) in the experimental group
Abdulah and Abdulla (2018)	KIDSCREEN-10 (parent) (Ravens-Sieberer et al., 2010)	Experimental group: significantly more physically active and energetic; less depressed, emotional, and stressed; more enjoyment of social/leisure time and more social participation; improved relationships and better health (all *p* < 0.05)
**Children with cancer: music media**		
Robb (2000)	Time sampling observations and coding of (i) behavior frequency (activity, attention, choice making, following directions, affective state) and (ii) environment support (verbal directions, activities, choices, positive non/verbal reinforcement, changes initiated by child, positive adult-initiated interaction, attention from adult) Affective Face Scale (McGrath, 1985)	Higher environmental support during music condition Significant main effects for environment (*p* < 0.001) and condition (*p* < 0.001) Significant interaction of condition × environment (*p* < 0.001) Higher behavior scores in music conditionSignificant main effects for behavior (*p* < 0.001) and condition (*p* < 0.001) Significant interaction of condition × behavior (*p* = 0.001) Significant correlation of environment and behavior: control (*p* < 0.001) and music (*p* = 0.002)
Barrera et al. (2002)	Adapted FACES pain scale (Bieri et al., 1990) Play Performance Scale (Lansky et al., 1987) Satisfaction questionnaire (SQ)	Improved child-reported feelings pre/post (*p* < 0.01) and higher for AME than passively engaged (*p* < 0.01) More parent-reported play after active vs. passive music engagement (*p* < 0.01) and more improvement for adolescents (*p* < 0.05) Satisfaction with music intervention Children <5 years: “I liked the guitar”; children 6–10: “I like the silly songs”; adolescents: “It made my nausea go away” Parents: comforting to child (64%); reducing child anxiety (58%) and own anxiety (49%); comments: “takes their mind off their disease/treatments,” “helps children and parents feel less anxious” Staff comments: “The sessions were excellent.” “She has such a therapeutic effect on the children and families.” “I don't understand what she does.”
Colwell et al. (2005)	Piers Harris Children's Self-Concept Scale (Piers and Herzberg, 2002)	*Significant improvements: pre–post-intervention* All subjects: total score (*p* = 0.004)Art: total score (*p* = 0.002) and popularity (*p* = 0.009) Music: school status (*p* = 0.02); physical appearance/attributes (*p* = 0.026) *Significant improvements: between-group differences* Music: greater intellectual/school status (*p* = 0.017) Art group: popularity (*p* = 0.021)
Robb et al. (2008)	Behavioral coding of coping behavior: facial affect, active engagement, initiation	AME: significantly more coping behaviors than ML or ASB Significantly higher positive facial affect and active engagement in AME than ML and ASB (*p* < 0.0001) Initiation significantly higher during AME than ASB (*p* < 0.05)
Giordano et al. (2020)	Modified Yale Pre-operative Anxiety Scale (Jenkins et al., 2014) Interviews with medical staff	Lower anxiety levels in music therapy group >90% medical staff satisfied with music therapy
**Children with cancer: composite arts-based therapies (music, movement, and art) and virtual reality play therapy**		
Madden et al. (2010)	Pediatric Oncology Quality of Life Inventory (Varni et al., 1998) SQ Faces Scale (McGrath et al., 1996) Emotional Reactions Checklist (Reid et al., 1998)	Randomized phase: parent-reported reduced pain (*p* = 0.03) and nausea (*p* = 0.006) Cohort phase: child-reported improved mood (*p* = 0.006): more excited (*p* = 0.04), happier (*p* = 0.02), less nervous (*p* = 0.02) Positive parent (e.g., “Really good” and “He was able to express feelings and creativity”) and provider satisfaction (e.g., “on a busy day it is chaotic if there are drums”)
Li et al. (2011)	Short State Anxiety Scale for Children (Li and Lopez, 2007) Center for Epidemiologic Studies Depression Scale for Children (Weissman et al., 1980)	Significantly less depression symptoms in intervention group (*p* = 0.02)
**Children with blood disorders and other health conditions: visual media**		
Beebe et al. (2010)	Pediatric Quality of Life Asthma Module (Varni et al., 2004) Beck Youth Inventory (Beck et al., 2005) Formal Elements Art Therapy Rating Scale : Draw a Person Picking an Apple from a Tree (Gantt and Tabone, 2003)	Post-intervention and 6 months: improved parent/child-reported quality of life and worry (all *p* < 0.05) Post-intervention: improved self-concept and anxiety; improved anxiety sustained for 6 months (all *p* < 0.05) Improved coping and resourcefulness at post-intervention and 6 months (all *p* < 0.05)
Stafstrom et al. (2012)	Childhood Attitude Toward Illness Scale (CATIS) (Austin and Huberty, 1993)	No differences pre/post-intervention Children and parents positive about art therapy
MacDonald et al. (2019)	Medical Outcomes Study Social Support scale (Sherbourne and Stewart, 1991) Mental Health Continuum Short-Form scale (Lamers et al., 2011) Problem Areas in Diabetes (Polonsky et al., 1995) SQ	29% mental health pre/post-intervention change: languishing/moderate 57% reduced diabetes distress post-intervention 80% intervention acceptable and effective
**Children with blood disorders and other conditions: music media and play therapy**		
Colwell et al. (2013)	Wong–Baker FACES Pain Rating Scale (Wong and Baker, 2001) State-Trait Anxiety Inventory for Children (Spielberger, 1973) Physiological measures Time sampling engagement/interaction	Intervention group more eye contact with therapist (*p* = 0.000)
Robb et al. (2014)	Measures related to Haase's Resilience in Illness Model	Post-intervention: TMV group (n = 41) significantly better courageous coping (*p* = 0.03) 100 days post-transplant: TMV group (n = 30) significantly better social integration (*p* = 0.028) and family environment (*p* = 0.008)
Tomaj et al. (2016)	Piers Harris Self Concept Scale (Piers and Herzberg, 2002)	Intervention group, significantly higher self-concept pre/post-intervention and 1 month FU (*p* < 0.001)
Uggla et al. (2018)	Pediatric Quality of Life Inventory (generic and cancer module) (Varni et al., 2002) Astrid Lindgren Children's Hospital Pain Scale (Lundqvist et al., 2014)	Music group: higher physical function post-intervention (*p* = 0.04); wait-list control group improved in all domains (*p =* 0.015)

*SR, self-report; SD, standard deviation; FU, follow-up; AME, active music engagement; ML, music listening; ASB, audio-storybooks; TMV, therapeutic music video*.

Eight studies were conducted in the USA (Robb, 2000; Colwell et al., 2005, 2013; Robb et al., 2008, 2014; Beebe et al., 2010; Madden et al., 2010; Stafstrom et al., 2012), two studies were carried out in Canada (Barrera et al., 2002; MacDonald et al., 2019), one in Iraq (Abdulah and Abdulla, 2018), one in Hong Kong (Li et al., 2011), two in Iran (Khodabakhshi Koolaee et al., 2016; Tomaj et al., 2016), one in Italy (Giordano et al., 2020), and one study in Sweden (Uggla et al., 2018).

### Quality of Studies Included in This Review

**Figure 2:**
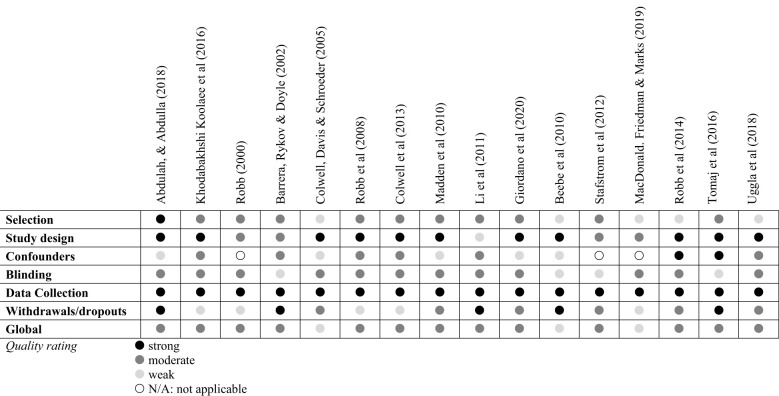
Quality assessment of studies included in the review.

**Figure 3:**
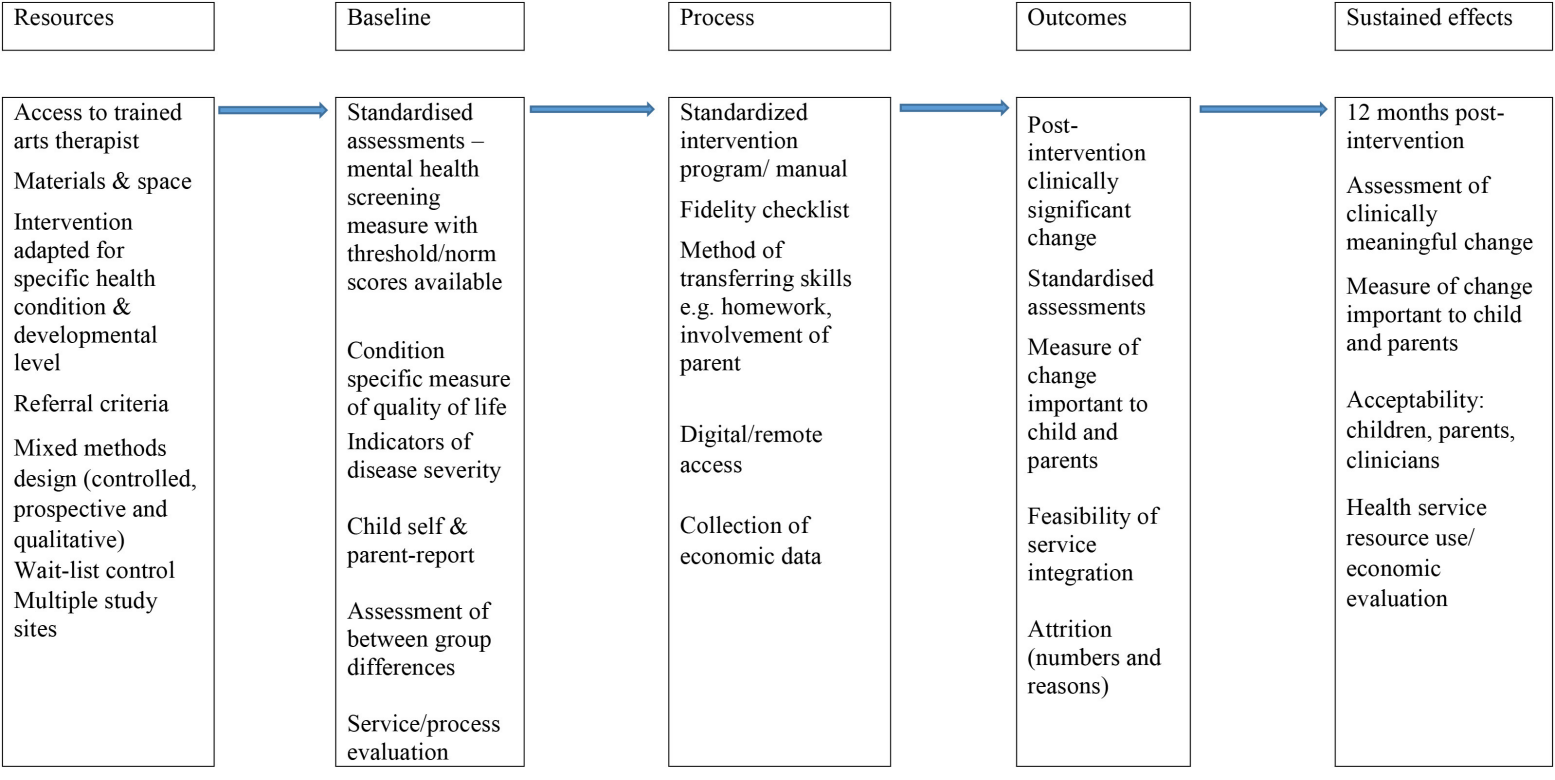
Conceptual model of characteristics important for future research trials evaluating effectiveness of arts-based therapies for mental health and well-being in pediatric physical health settings.

The quality of studies was appraised using structured criteria relevant for intervention effectiveness evaluations, and Figure 2 shows a summary of the quality assessments (Thomas et al., 2004). None of the studies had global ratings of high quality, 13 studies had a moderate global rating, and 3 studies had weak ratings of quality. All studies had some strong components (e.g., describing participant randomization, use of a published outcome measure); however, the global quality rating of all articles was reduced by specific design limitations. These included confounding variables, a narrow recruitment pool (e.g., one inpatient unit), or a lack of blinding. The purpose of the quality appraisal was to identify areas to address when designing a future trial, and potential bias in the studies reviewed is integrated into the narrative synthesis below and informs the model in Figure 3.

### Narrative Synthesis

Given that evidence of the effectiveness of psychological interventions for individual pediatric physical health conditions is limited (Moore et al., 2019), we present the results according to health condition.

### Arts-Based Therapies for Children With Cancer

Nine studies evaluated arts-based therapies for children with cancer. Six studies had controlled designs, three of those describing a randomization process and the remainder having cohort designs.

#### Children With Cancer: Arts-Based Therapies Using Visual Media

Two studies evaluated visual/craft media (Khodabakhshi Koolaee et al., 2016; Abdulah and Abdulla, 2018). In the first study, parent-reported health-related quality of life measured using a short (10-item) questionnaire improved after a group intervention, though a limitation of the study was no measurement of child-reported outcomes (Abdulah and Abdulla, 2018). The authors stated that the intervention was not provided during chemotherapy, and this level of detail is important information for replication in future research and practice. The second study using visual media demonstrated significantly more reductions in anger and anxiety for the intervention group; a strength of the study was the specific inclusion criteria (scoring above the mean on standardized measures of anxiety and anger) and provision of an intervention schedule (Khodabakhshi Koolaee et al., 2016). The study demonstrated statistically significant post-intervention improvements but did not report whether changes were clinically significant. Both studies recruited narrow age groups relative to other studies reviewed, and this is a strength given developmental differences, though scant detail on intervention components or where to find detailed intervention schedules was a limitation.

#### Children With Cancer: Arts-Based Therapies Using Music

Five studies evaluated the effectiveness of music-based therapies for children with cancer (Robb, 2000; Barrera et al., 2002; Colwell et al., 2005; Robb et al., 2008; Giordano et al., 2020). The first study provided a detailed protocol and demonstrated that music elicited significantly more engaging behavior but no improvements on an outcome measure of affect (Robb, 2000). Music-based therapy was associated with improvements in feelings (e.g., “It made my nausea go away”) for actively involved Canadian pediatric hematology inpatients (Barrera et al., 2002). A limitation was the single-group single-site design, while a strength was the use of child- and parent-reported outcome measures and qualitative assessment of acceptability (Barrera et al., 2002). A third study evaluating music-based therapy for children with cancer recruited from an inpatient service demonstrated significant improvements in self-concept, though the control group had art activities, which may have been a confounder (Colwell et al., 2005). Strengths of the study were the use of a board-certified therapist and a computer music program, suggesting potential for replication. Giordano et al. (2020) evaluated pre-operative music therapy for children with leukemia and parents and found less anxiety relative to a control group, and acceptability to medical staff was high. Finally Robb et al. (2008) demonstrated significantly more positive coping behaviors including smiling and active engagement in music group participants compared to two control groups. Although published outcome measures were not used, the study was included in the review because outcomes were assessed using a formalized behavioral rating system with integrated reliability checks. A strength of the study was the multi-site randomized controlled trial (RCT) design and the use of intervention delivery guides.

#### Children With Cancer: Composite Arts-Based Therapies (Music, Movement, and Art) and Virtual Reality Play Therapy

One study evaluating a composite arts-based intervention (music, movement, and art) described very specific participant inclusion criteria—those with a brain tumor, receiving treatment for at least 3 months, no less than weekly (Madden et al., 2010). The development of psychological interventions for children with specific physical health conditions has been recommended (Moore et al., 2019). This is therefore a strength of the study, compared to other studies reviewed recruiting children with a heterogeneity of conditions. A limitation was the wide age range of participants, while another study strength was a mixed methods design, including qualitative perspectives of service providers. Service provider perspectives were positive, while child-reported mood and parent-reported pain improved significantly (Madden et al., 2010).

One study evaluated virtual reality play therapy using published measures of anxiety and depression (Li et al., 2011). This is a study strength in relation to other included studies, as limited availability of reliable psychological interventions specifically for anxiety and depression in children with physical health LTCs has been described (Thabrew et al., 2018). There were significantly more reductions in depression symptoms in the intervention group (Li et al., 2011).

In summary, nine studies examined the effectiveness of arts-based therapies on the mental well-being of children undergoing treatment for cancer. Overall, the studies indicated some positive impact; however, the heterogeneity of intervention content and implementation, the outcomes measured, and limitations regarding study quality (e.g., presence of confounding variables, lack of randomization, and single-site evaluations) make it difficult to draw firm conclusions and generalizations or to replicate studies.

### Arts-Based Therapies for Children With Blood Disorders and Other Health Conditions

#### Children With Blood Disorders and Other Health Conditions: Arts-Based Therapies Using Visual Media

Three studies evaluated interventions for children with asthma, epilepsy, and insulin-dependent diabetes (Beebe et al., 2010; Stafstrom et al., 2012; MacDonald et al., 2019).

The first study using visual craft media demonstrated improved mood and quality of life in children with asthma, recruited from a school outpatient clinic (Beebe et al., 2010). Study strengths were measuring child- and parent-report perspectives, providing an intervention schedule, use of health condition–specific outcome measures, controlled design, and data collection 6 months post-intervention.

In the second study, 16 children with epilepsy reported positively on art therapy received; however, outcome measures showed no improvements (Stafstrom et al., 2012). Lastly, 80% of participants with diabetes rated arts-based therapies positively in a satisfaction survey; however, participants were required to attend weekly from remote/rural locations, and attrition was high (MacDonald et al., 2019).

#### Children With Blood Disorders and Other Health Conditions: Music Media and Play Therapy

Children with a range of health conditions receiving one-to-one music-based therapy demonstrated significant reductions in anxiety or pain but no more so than control groups (music listening/composition) (Colwell et al., 2013). A multi-site music therapy evaluation (n = 113) for children with hematopoietic stem cell transplants demonstrated social, family, and spiritual improvements 3 months post-intervention (Robb et al., 2014). A strength of the study was a process for assessing intervention fidelity across sites/therapists.

An RCT of music-based therapy for children receiving hematopoietic stem cell transplants showed improved physical function post-intervention. A study strength was describing parent involvement and the wait-list control design, so all participants had access to the intervention (Uggla et al., 2018).

Finally self-concept was improved 1 month post-intervention (eight play therapy sessions) for 60 children with thalassemia major across two hospital sites (Tomaj et al., 2016).

In summary, seven studies examined the effectiveness of arts-based therapies on the mental well-being of children undergoing treatment for blood disorders and other health conditions. Once again, the studies indicated some positive impact, but significant heterogeneity of intervention characteristics (e.g., whether individual or group format), variety of outcomes measured, and design limitations mean that firm conclusions about effectiveness across studies are not possible.

## Discussion

The review identified and appraised 16 articles evaluating the effectiveness of arts-based therapies for improving the mental health of children with physical health LTCs. Some improvements were demonstrated in articles selected, including, for example, improved quality of life, coping behaviors, self-concept, improved mood, and reduced anxiety. Participants included children with a range of physical health LTCs, and interventions comprised arts-based therapies utilizing music, play, and visual media in the context of a therapeutic relationship. However, the heterogeneity in intervention characteristics and design limitations identified mean that it is not possible to make conclusions about effectiveness across studies. Further, the review highlights that the existing evidence base for the effectiveness of arts-based therapies as a psychological intervention for pediatric physical health settings is sparse and requires development (Cohen-Yatziv and Regev, 2019).

A lack of post-intervention positive changes on published outcome measures, in some studies, contrasted with measures of satisfaction (where used), which did indicate benefits. This is in accordance with the findings of a recent evidence synthesis of mental health interventions for children with physical health LTCs, which found meta-ethnographic evidence of benefit but a lack of effectiveness evidence (Moore et al., 2019). This underlines the importance of measuring outcomes using quantitative and qualitative methods across a range of indicators (e.g., school attendance) and from a range of perspectives when designing future evaluations of arts-based therapies for children with physical health LTCs. The strengths and limitations of the studies reviewed informed a logic model (Figure 3), and we use this to make suggestions to guide the design of future research evaluations of the effectiveness of arts-based therapies for children with physical health LTCs in order to help develop the field (Cohen-Yatziv and Regev, 2019). For example, none of the studies reviewed assessed if changes reached minimum thresholds for clinically significant or meaningful change (de Vet et al., 2006); further, most of the effectiveness evaluations in the studies reviewed are cross-sectional. In future studies, quantitative prospective assessments would facilitate evaluating sustained effects after the intervention, and the use of outcome measures with published norm/cut-scores would facilitate more accurate interpretation of any changes in scores resulting from interventions. Most studies recruited participants from a single site. Future research recruiting from multiple sites should incorporate processes for ensuring intervention consistency between sites, evaluation of intervention effectiveness across sites, and feasibility of sustainable integration of arts-based therapies into pediatric physical health LTC service structures, including identification of barriers and facilitators. Most of the studies were conducted in the USA and generalizability to the UK NHS or health services in other countries cannot be assumed, given different health service structures and funding.

## Review Strengths and Limitations

Limitations of the review include small numbers of studies identified and heterogeneity in their design, so quantitative pooling of results across studies or health conditions was not possible. Arts-based therapies may require tailoring for children with different physical health LTCs; however, an in-depth synthesis of findings on the effectiveness of arts-based therapies for specific health conditions was not possible.

We have taken a reductionist perspective of effectiveness; our inclusion criterion was studies using published mental health outcome measures, and we excluded qualitative studies. We acknowledge that this definition of effectiveness will not capture all effects, mechanisms, and dynamic processes of change occurring during arts-based therapies (Gerber et al., 2018). We did not screen gray literature or include end-user consultation, and we did not have resources to complete full independent screening and quality appraisals or translate and include articles not published in English. These are potential sources of bias in our review.

The aim of the review was to explore the effectiveness of arts-based therapies to improve mental health for children with physical health LTCs. Only one RCT of an arts-based therapy (music) was identified in a recent review of psychological interventions for children with physical health LTCs (Moore et al., 2019). Given this, we looked broadly and included cohort and within-subject designs, which are, however, not strong indicators of effectiveness. Elevated mental health symptoms were not an inclusion criterion for our review, and only one study (Khodabakhshi Koolaee et al., 2016) recruited participants scoring above the mean on standardized measures (of anxiety and anger); this is a limitation of our review with regard to assessing improved mental health. However, given the long wait times for access to child mental health services, it is important to explore the effectiveness of reducing sub-threshold mental health symptoms and any preventative potential of arts-based therapies, and this is a strength of the review (NHS Digital, 2019).

## Study Implications and Future Research

The review identified a number of design limitations important to address in future research, and we have summarized these in a model. Future trials would benefit from including end-user consultation in intervention design and service integration and could evaluate the involvement of parents and online formats of delivery to support transferring acquired/internalized coping skills outside/after intervention and for children in remote locations.

The choice of evidence-based psychological interventions available for children with physical health LTCs is currently small (Moore et al., 2019). Arts-based therapies present a potential option; however, the findings from the review confirm that research is required prior to any sustainable integration into physical heath settings. The review has highlighted some areas to clarify in future research. None of the studies reviewed distinguished between using arts-based therapies as preventative or treatment interventions. Given health service resource constraints, it is unlikely that arts-based therapies can be available to all children in physical health LTC settings, so this distinction requires consideration in future studies. Future research should evaluate how arts-based therapies might be integrated into assessment processes to support clinical teams in identifying mental health difficulties and facilitate children accessing the support they require early. In future research, economic analysis could facilitate examining any savings made by avoiding treatment complications through providing arts-based therapies (Seid et al., 2004; Shaw, 2016). In the absence of any extra health service funding, economic evaluations including an invest-to-save analysis will be important to demonstrate any reduced costs from integrating arts-based therapies in the NHS, e.g., fewer referrals to child and adolescent mental health services (Shaw, 2016). Participant inclusion criteria were not always clear in the studies reviewed. Again, limited health service resources will influence decisions about access to psychological interventions, and identifying the characteristics of children who may benefit most from arts-based therapies is important to clarify in future research and could be explored using mixed methods approaches.

## Conclusions

Integration of psychological support into pediatric physical health settings is an indicator of service quality (DoH, 2011; Foster et al., 2017; Parsons et al., 2017; NHSE, 2018). However, there is limited evidence-based psychological support available for children with physical health LTCs, and this is a barrier to the provision of integrated services (Thabrew et al., 2018; Moore et al., 2019). The findings from this systematic review of effectiveness demonstrated that replication of interventions and outcomes across studies was absent, so conclusions about the effectiveness of arts-based therapies for improving the mental well-being of children with physical health LTCs cannot be made. The findings also highlight design characteristics important to incorporate when developing future trials evaluating the effectiveness of arts-based therapies. If future robustly designed research studies can demonstrate the effectiveness of arts-based therapies for children with physical health LTCs, commissioning is more likely, and this could potentially create increased choice of psychological interventions for children and families, be an alternative to talking-based therapies for children who might find it hard to speak about their difficulties, and increase the resources available for service providers.

## Data Availability Statement

All datasets presented in this study are included in the article/supplementary material.

## Author Contributions

SH, SW, PW, AZ, and SJ conceptualized the review. SW, AZ, and JB analyzed results. SW wrote the first draft of the manuscript. All authors contributed to editing, commenting, and revising manuscript versions, read and approved the submitted version.

## Conflict of Interest

The authors declare that the research was conducted in the absence of any commercial or financial relationships that could be construed as a potential conflict of interest.

## Appendix

**Table A1:** Search strategy used for the EBSCO interface and Medline database.

1.	(Art therap$ or art psychotherap$ or creative arts therap$). m_titl.	3,337
2.	Limit 1 to yr = “2000-Current”	2,662
3.	Drama therap$. m_titl.	231
4.	Limit 3 to yr = “2000-Current”	163
5.	(Music therap$ or music intervention or musical therap$).m_titl.	5,542
6.	Limit 5 to yr = “2000-Current”	4,736
7.	Play therap$.m_titl.	1,733
8.	Limit 7 to yr = “2000-Current”	1,285
9.	(Dance therap$ or movement therap$ or dmt).m_titl.	2,676
10.	Limit 9 to yr = “2000-Current”	2,397
11.	2 or 4 or 6 or 8 or 10	11,218
12.	(Child$ or adolescent$ or youth$ or teenager$).m_titl.	1,937,337
13.	Limit 12 to yr = “2000-Current”	1,450,570
14.	(Pediatric or child$ or adolescent$).m_titl.	2,138,347
15.	Limit 14 to yr = “2000-Current”	1,619,826
16.	13 or 15	1,698,497
17.	11 and 16	2,038
